# Bulk‐Plasmon‐Mediated Free‐Electron Radiation Beyond the Conventional Formation Time

**DOI:** 10.1002/advs.202300760

**Published:** 2023-05-01

**Authors:** Fuyang Tay, Xiao Lin, Xihang Shi, Hongsheng Chen, Ido Kaminer, Baile Zhang

**Affiliations:** ^1^ Department of Electrical and Computer Engineering Rice University Houston TX 77005 USA; ^2^ Applied Physics Graduate Program Smalley–Curl Institute Rice University Houston TX 77005 USA; ^3^ Interdisciplinary Center for Quantum Information State Key Laboratory of Modern Optical Instrumentation ZJU‐Hangzhou Global Science and Technology Innovation Center College of Information Science and Electronic Engineering Zhejiang University Hangzhou 310027 China; ^4^ International Joint Innovation Center the Electromagnetics Academy at Zhejiang University Zhejiang University Haining 314400 China; ^5^ Department of Electrical Engineering Technion‐Israel Institute of Technology Haifa 32000 Israel; ^6^ Jinhua Institute of Zhejiang University Zhejiang University Jinhua 321099 China; ^7^ Division of Physics and Applied Physics School of Physical and Mathematical Sciences Nanyang Technological University Singapore 637371 Singapore; ^8^ Centre for Disruptive Photonic Technologies Nanyang Technological University Singapore 637371 Singapore

**Keywords:** Ferrell radiation, free‐electron radiation, plasmonics, transition radiation

## Abstract

Free‐electron radiation is a fundamental photon emission process that is induced by fast‐moving electrons interacting with optical media. Historically, it has been understood that, just like any other photon emission process, free‐electron radiation must be constrained within a finite time interval known as the “formation time,” whose concept is applicable to both Cherenkov radiation and transition radiation, the two basic mechanisms describing radiation from a bulk medium and from an interface, respectively. Here, this work reveals an alternative mechanism of free‐electron radiation far beyond the previously defined formation time. It occurs when a fast electron crosses the interface between vacuum and a plasmonic medium supporting bulk plasmons. While emitted continuously from the crossing point on the interface—thus consistent with the features of transition radiation—the extra radiation beyond the conventional formation time is supported by a long tail of bulk plasmons following the electron's trajectory deep into the plasmonic medium. Such a plasmonic tail mixes surface and bulk effects, and provides a sustained channel for electron–interface interaction. These results also settle the historical debate in Ferrell radiation, regarding whether it is a surface or bulk effect, from transition radiation or plasmonic oscillation.

## Graphical abstract

This work reveals a new type of free‐electron radiation that occurs far beyond the conventional formation time and is mediated by a long tail of bulk plasmons. Different from Cherenkov radiation and transition radiation, this plasmonic tail mixes surface and bulk effects and provides a sustained channel for electron–interface interaction. The bulk‐plasmon‐mediated free‐electron radiation settles the historical debate in Ferrell radiation.

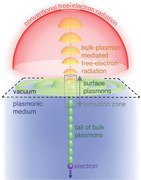

## 1 Introduction

The interaction between fast‐moving electrons and optical media causes free‐electron radiation. A famous example is Cherenkov radiation,^[^ ^1^, ^2^, ^3^, ^4^, ^5^, ^6^ ^]^ in which photons are emitted from the bulk of a medium when the electron's speed exceeds the speed of light in the medium. Another type of free‐electron radiation, known as transition radiation,^[^ ^7^, ^8^, ^9^, ^10^, ^11^, ^12^, ^13^, ^14^ ^]^ refers to photon emission from an interface—when an electron crosses an interface between different media, the electron will always emit photons, at any speed. As an alternative photon emission mechanism apart from atomic spontaneous emission and stimulated emission, free‐electron radiation plays a significant role in many practical applications, ranging from high‐energy particle detectors, free‐electron lasers, electron microscopies, medical imaging, security scanning, to astronomy and cosmology.^[^ ^15^, ^16^, ^17^, ^18^, ^19^, ^20^, ^21^ ^]^

In any type of radiation, it takes time for photons to be emitted, and the time interval is called the formation time. In the context of free‐electron radiation, the concept of formation time in a bulk medium was firstly proposed by Ter‐Mikaelian in Landau's seminar in 1952,^[^ ^22^, ^23^ ^]^ and then further developed by Landau himself,^[^ ^24^, ^25^ ^]^ with its physical effects experimentally demonstrated in 1990s.^[^ ^26^, ^27^, ^28^ ^]^ Later, Ginzburg extended the formation time concept into photon emission from an interface, namely transition radiation.^[^ ^8^, ^9^, ^29^ ^]^ As defined by Ginzburg, photons in transition radiation are emitted within one formation time.^[^ ^9^ ^]^ This concept has already provided valuable guidance for practical applications. For example, the influence of formation time should be avoided in the design of transition radiation detectors,^[^ ^10^ ^]^ which are widely used in the identification of high‐energy particles.

**Figure 1:**
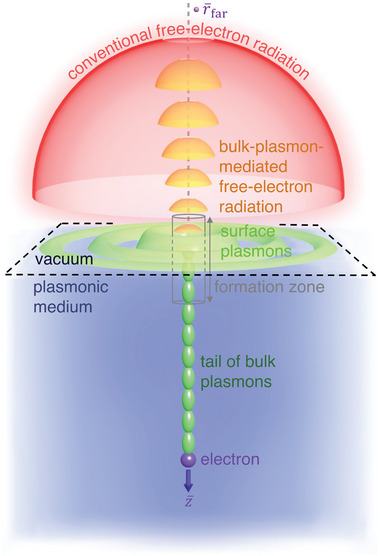
Schematic of the bulk‐plasmon‐mediated free‐electron radiation beyond the conventional formation time. A fast‐moving electron with velocity $\bar{v}=\hat{z}v$ impinges on an interface and induces radiation, where *v* = 0.4*c* is used. The interface separates regions 1 (namely vacuum) and region 2 (a dielectric or a plasmonic medium), whose relative permittivity is *ε*_1r_ and *ε*_2r_, respectively. The radiation beyond the conventional formation time occurs due to the formation of a long tail of bulk plasmons when the electron moves inside the plasmonic medium, whose optical response is described by a Drude‐like formula, namely $\varepsilon_{\mathrm{Drude}}\left(\omega \right)=1-\frac{\omega_{p}^{2}}{\omega^{2}+i\omega /\tau }$.

Here we reveal an alternative mechanism of free‐electron radiation far beyond the previously defined formation time. It can occur when a fast‐moving electron crosses the interface between free space and a plasmonic medium supporting bulk plasmons, such as metals at the plasma frequency (**Figure** **1**). This radiation is supported by a long tail of bulk plasmons following the electron's trajectory deep into the plasmonic medium (see Movies S1–S4 (Supporting Information) for a demonstration). Such a plasmonic tail mixes the bulk and surface effects, making the radiation neither a pure bulk effect as in Cherenkov radiation, nor a pure surface effect as in transition radiation. A striking feature for this bulk‐plasmon‐mediated radiation, as we will demonstrate later, is its long duration far beyond the formation time historically defined for free‐electron radiation.

It is interesting to note that such a geometry of electrons bombarding plasmonic media has been long studied in the field of plasmonics since 1950s. In fact, the existence of surface plasmons was firstly confirmed with electrons bombarding a metal film.^[^ ^30^ ^]^ However, the complex electron–photon–plasmon interaction has left a few issues in the history that have not been fully resolved. A typical example is the Ferrell radiation^[^ ^31^, ^32^ ^]^ (i.e., the enhanced radiation at the plasma frequency of the bulk medium), which could be ascribed to a surface effect with pure transition radiation, or a bulk effect induced by plasmonic oscillation, or both, but so far there is no decisive conclusion^[^ ^33^, ^34^, ^35^, ^36^, ^37^, ^38^, ^39^, ^40^ ^]^ (see Section S5 (Supporting Information) for a historical survey). With our revealed mechanism of bulk‐plasmon‐mediated free‐electron radiation beyond the conventional formation time, it becomes feasible to settle this historical debate here.

## 2 Results and Discussion

**Figure 2:**
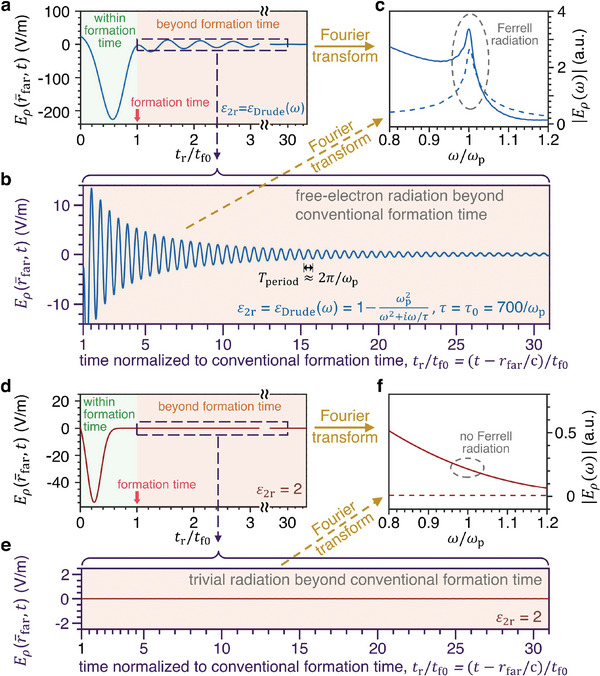
Representation in the time domain of the bulk‐plasmon‐mediated free‐electron radiation beyond the conventional formation time. The structural setup is the same as Figure 1. a) Temporal evolution of the backward‐radiation field $E_{\rho }\left({\bar{r}}_{\mathrm{far}},t\right)$ at a point ${\bar{r}}_{\mathrm{far}}\;$far away from the interface but close to the electron trajectory when region 2 is filled by a plasmonic medium with *ε*_2r_ = *ε*_Drude_(*ω*). Here and below, we set *t* = 0 as the moment when the electron enters the formation zone in vacuum, *t*_r_ = *t* − *r*_far_/*c* as the retarded time, $r_{\mathrm{far}}=\left|{\bar{r}}_{\mathrm{far}}\right|$, *t*_f0_ = *t*_f_ (*ω* = *ω*_p_), and *t*_f_(*ω*) is the conventional formation time defined for transition radiation. b) An enlarged plot of the radiation field beyond the conventional formation time (highlighted by a purple dashed square in (a)). c) Frequency‐domain analysis of the radiation in different ranges of time. The solid line corresponds to the Fourier transform of radiation from *t*/*t*_f0_ = 0 to *t*/*t*_f0_ = 40 in (a), while the dashed line corresponds to the Fourier transform of radiation from *t*/*t*_f0_ = 1 to *t*/*t*_f0_ = 40 in (b). d–f) Conventional free‐electron radiation when region 2 is filled by a regular dielectric with *ε*_2r_ = 2. All analysis in (d–f) is the same as (a–c).

We consider in Figure 1 that a fast electron moves with a velocity $\bar{v}=\hat{z}v$ and penetrates the interface separating region 1 and region 2, where their relative permittivity is *ε*_1r_ = 1 (free space) and *ε*_2r_ (either a dielectric or a plasmonic medium), respectively, *v* = 0.4*c*, and *c* is the speed of light in free space. By imposing the constraint of *ε*_2r_ < (*c*/*v*)^2^ = 6.25, we can exclude the possibility of Cherenkov radiation, since the electron's speed falls below the Cherenkov threshold. Consequently, the only possible radiation, according to conventional theories of classical electrodynamics, should be transition radiation, as studied by Ginzburg and many other colleagues.^[^ ^9^, ^12^, ^29^, ^41^ ^]^ Since we are interested in the backward radiation propagating almost parallel to the electron's trajectory, the conventional formation time of transition radiation, as defined by Ginzburg, is $t_{f}\left(\omega \right)=\frac{2\pi }{\omega |1+\frac{v}{c}\sqrt{\varepsilon_{1r}}|}+\frac{2\pi }{\omega |1-\frac{v}{c}\sqrt{\varepsilon_{2r}}|}$,^[^ ^8^, ^9^ ^]^ where *ω* is the angular frequency; see section S2 in Supporting Information. To facilitate the discussion, *t*_f0_ = *t*_f_(*ω*_p_) is chosen as a reference to normalize the horizontal coordinate of time in **Figure** **2**, where *ω*_p_ is the plasma frequency of plasmonic media (e.g., metals). Accordingly, the length of formation zone, also known as the formation length or coherence length,^[^ ^8^, ^22^, ^23^, ^42^ ^]^ is *L*_f0_ = *vt*_f0_.

Now we consider a plasmonic medium in region 2 with *ε*_2r_ = *ε*_Drude_(*ω*). To capture the role of losses, we employ a Drude‐like formula to describe the relative permittivity of plasmonic media, namely$\;\varepsilon_{\mathrm{Drude}}\left(\omega \right)=1-\frac{\omega_{p}^{2}}{\omega^{2}+i\omega /\tau }$, where the plasma frequency is set to be *ω*_p_ = 13.9 petahertz, and *τ* is the relaxation time; note that the nonlocal response of plasmonic media has a minor influence on the radiation revealed here; see Figures S7 and S8 (Supporting Information). As a comparison, we also consider the control situation when region 2 is a regular dielectric with *ε*_2r_ = 2.

Figure 2a–f shows the temporal evolution of the backward‐radiation field at a point ${\bar{r}}_{\mathrm{far}}$ far away from the interface but close to the electron's trajectory, where the angle between ${\bar{r}}_{\mathrm{far}}$ and $-\bar{z}$ is *θ*_far_ ≃ 7°. The major part of emitted photons at *θ*_far_ is formed within *t*_f0_, as shown in Figure 2a,d. If the fast electron is within the conventional formation zone of transition radiation, according to Ginzburg's analysis, the electron can directly interact with the interface. Then the significant process of photon emission within the conventional formation time in Figure 2a,d can be treated as a surface effect. Beyond *t*_f0_, if region 2 is a plasmonic medium with *ε*_2r_ = *ε*_Drude_(*ω*), there are sizable radiation fields that oscillate periodically far beyond *t*_f0_ in Figure 2a,b, where the relaxation time *τ* = *τ*_0_ is used and *τ*_0_ = 700/*ω*_p_. In contrast, if region 2 is a dielectric with *ε*_2r_ = 2, the radiation field beyond *t*_f0_ is negligible as shown in Figure 2d,e.

Moreover, the radiation fields beyond the conventional formation time *t*_f0_ in Figure 2b have their frequency close to the plasma frequency. Figure 2c shows that after the electron crosses the interface, the radiation peak around the plasma frequency is mainly contributed by the emission process of photons beyond the conventional formation time *t*_f_(*ω* = *ω*_p_) = *t*_f0_.

**Figure 3:**
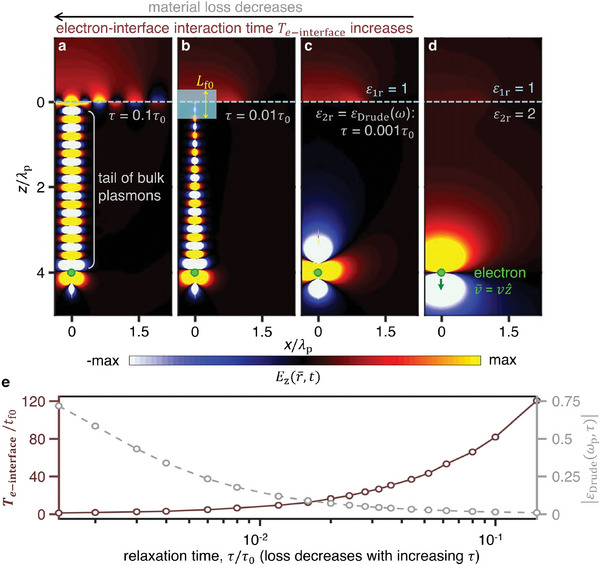
Spacetime‐domain illustration of the underlying physics for the bulk‐plasmon‐mediated free‐electron radiation. This radiation is caused by the indirect electron–interface interaction, which is mediated via a long tail of bulk plasmons and can exist far beyond the conventional formation time. a–d) Spatial distribution of the electric field $E_{z}\left(\bar{r},t\right)$; see also in Movies S1–S4 (Supporting Information). e) Electron–interface interaction time *T*_*e* − interface_ as a function of the relaxation time *τ*. *T*_*e* − interface_ can far exceed *t*_f0_, if *τ* increases or if |*ε*_Drude_(*ω*_p_,*τ*)| (instead of merely Im(*ε*_Drude_(*ω*_p_,*τ*))) decreases down to a minor value. The shaded region at the interface in (b) represents the formation length of transition radiation, *L*_f0_. Besides, *λ*_p_ = 2*πc*/*ω*_p_.

**Figure** **3**a–d shows that the revealed extra radiation beyond *t*_f0_ is caused by the interaction between the interface and a long tail of excited bulk plasmons (both the transverse and longitudinal electromagnetic waves are considered for bulk plasmons; see Section S7, Supporting Information). The reason is that the tail of excited bulk plasmons in the plasmonic medium not only follows the electron's trajectory but also can attach to (and thus interact with) the interface for a long time (Figure 3a). In other words, even when the fast electron is far beyond the formation zone of transition radiation, the electron will continue to interact with the interface at the same crossing point; this way, the radiation process is unfinished beyond the conventional formation time, and the fast electron will continue to emit photons from the interface (Figure 3a). Since the excitation of bulk plasmons is a bulk response of the plasmonic medium, it is reasonable to treat the formation of the radiation beyond *t*_f0_ as a mixture of surface‐bulk effect, instead of solely a surface effect. Besides, since the surface plasmons at the surface of plasmonic media exist far below the plasma frequency *ω*_p_ (i.e., $\omega <\omega_{p}/\sqrt{2}$) but the excited bulk plasmons have their frequency close to *ω*_p_, the surface plasmons cannot be excited by the bulk plasmons but can be excited only by the direct electron–interface interaction within the conventional formation time;^[^ ^29^ ^]^ see more discussion about Figure 3a–d in Section S4, Supporting Information. This way, we denote the radiation beyond *t*_f0_ as the bulk‐plasmon‐mediated free‐electron radiation, while denoting the radiation within *t*_f0_ as the conventional free‐electron radiation (or transition radiation).

Due to the interface‐bulk effect, we shall redefine the formation time for this bulk‐plasmon‐mediated free‐electron radiation. This formation time, labelled as *T*_*e* − interface_, describes the electron–interface interaction time and is here defined as the time taken for the tail of bulk plasmons to leave the interface, which can be treated simply as the ratio between the full length of the tail (created by a fast electron moving inside a homogeneous plasmonic medium) and the electron's velocity. Apparently, this formation time *T*_*e* − interface_ is highly dependent on the loss of plasmonic media. For example, *T*_*e* − interface_ > 100*t*_f0_ if *τ* > 0.1*τ*_0_, as shown in Figure 3e. The large value of *T*_*e* − interface_ directly indicates that the bulk‐plasmon‐mediated free‐electron radiation can occur far beyond the conventional formation time of transition radiation. This bulk‐plasmon‐mediated free‐electron radiation is distinct from the conventional free‐electron radiation trapped by resonators (inside which the outcoupling process of the already‐formed photons can last for a relatively‐long time without the existence of electron–resonator interactions, if the quality factor of resonators is high).^[^ ^43^ ^]^.

**Figure 4:**
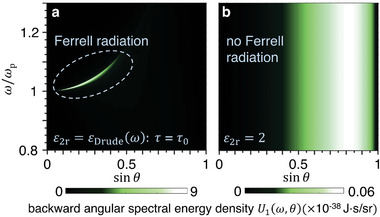
Frequency‐domain analysis of the bulk‐plasmon‐mediated free‐electron radiation. The structural setup is the same as Figure 1. a,b) Angular spectral energy density *U*_1_(*ω*,*θ*) of the backward radiation. Region 2 is filled by a plasmonic medium with *ε*_2r_ = *ε*_Drude_(*ω*) in (a) and a regular dielectric with *ε*_2r_ = 2 in (b).

To further understand the bulk‐plasmon‐mediated radiation, we explore it in the frequency domain in **Figure** **4**. Figure 4a,b shows the angular spectral energy density *U*_1_(*ω*,*θ*) of backward radiation. From Figure 4a, there is a radiation peak near the plasma frequency if region 2 is the plasmonic medium. This radiation peak is historically known as the Ferrell radiation,^[^ ^11^, ^31^, ^32^, ^33^, ^34^, ^35^, ^36^, ^37^, ^38^, ^39^, ^40^, ^44^ ^]^ which has caused a long debate about its physical origin, regarding whether it is a surface or bulk effect, from transition radiation or plasmonic oscillation (see Section S5 (Supporting Information) for a detailed histrocial survey). Nowadays, most literatures have ascribed Ferrell radiation to radiative surface plasmons or the so‐called Ferrell mode,^[^ ^32^, ^38^, ^40^, ^44^ ^]^ but a complete picture is still lacking to fully settle the debate.

We find that the introduction of the bulk‐plasmon‐mediated free‐electron radiation beyond the conventional formation time can address this issue. Firstly, there are two types of plasmonic oscillations simultaneously, bulk plasmons in the bulk and radiative surface plasmons on the interface. While bulk plasmons are excited as the plasmonic tail deep into the plasmonic medium, the tail also touches the interface, exciting radiative surface plasmons. Secondly, while it is the radiative surface plasmons that directly emit photons from the interface (but that alone cannot continuously emit photons far beyond the conventional formation time due to their large decay rate), it is the bulk plasmons that provide the energy, and the bulk plasmons in turn draw energy from the fast‐moving electron. Along the way, the electromagnetic field extends coherently from the fast‐moving electron to the interface. In other words, it takes a long distance, and thus a long time interval, to “peel off” the electromagnetic field from the electron, eventually forming photons. Thirdly, the Ferrell radiation is contributed by both transition radiation and plasmonic oscillation. These two contributions are impossible to be distinguished in the frequency domain but can be separated in the time domain. The transition radiation, as described by Ginzburg, occurs only within one conventional formation time. The significant radiation beyond the conventional formation time comes from the plasmonic oscillation, as we have analyzed in Figure 2.

## 3 Conclusion

In conclusion, we have revealed the emergence of bulk‐plasmon‐mediated free‐electron radiation beyond the conventional formation time by investigating the penetration of a fast‐moving electron through the interface of a plasmonic medium. This extra radiation is closely related to a long tail of bulk plasmons, which follows the electron's trajectory deep into the plasmonic medium and can attach to and thus interact with the interface for a very long time. Correspondingly, this tail provides a unique route to mix the surface and bulk effects, significantly extend the electron–interface interaction, and then create light emission far beyond the conventional formation time. Therefore, the revealed bulk‐plasmon‐mediated free‐electron radiation is intrinsically a mixture of surface‐bulk effect, distinct from Cherenkov radiation as purely a bulk effect or transition radiation as purely a surface effect. Our finding also provides a new perspective for Ferrell radiation and settles its historical debate regarding its physical origin. The bulk‐plasmon‐mediated free‐electron radiation may further trigger many open questions, concerning, for example, the observation of the long tail of bulk plasmons or the radiation beyond the conventional formation time, the possibility to largely enhance particle–light–matter interactions and the design of advanced light sources by exploiting bulk plasmons in other types of free‐electron radiation such as Smith‐Purcell radiation and synchrotron radiation.

## Conflict of Interest

The authors declare no conflict of interest.

## Supporting information

Supporting Information

Supplemental Movie 1

Supplemental Movie 2

Supplemental Movie 3

Supplemental Movie 4

Supplemental Movie 5

## Data Availability

The data that support the findings of this study are available from the corresponding author upon reasonable request.
