# Supplementary material for: Bulk‐Plasmon‐Mediated Free‐Electron Radiation Beyond the Conventional Formation Time
Source: Adv Sci (Weinh). 2023 May 1;10(20):2300760. doi: 10.1002/advs.202300760 (PMC10369295; doi:10.1002/advs.202300760)
Supplement: Supplementary file 1 — Supporting Information [file ADVS-10-2300760-s005.pdf]

ADVANCED SCIENCE
Open Access

Supporting Information

for *Adv. Sci.*, DOI 10.1002/advs.202300760

Bulk-Plasmon-Mediated Free-Electron Radiation Beyond the Conventional Formation Time

Fuyang Tay, Xiao Lin, Xihang Shi, Hongsheng Chen, Ido Kaminer* and Baile Zhang**

Supporting Information

Bulk-plasmon-mediated free-electron radiation beyond the conventional formation time

Fuyang Tay, Xiao Lin*, Xihang Shi, Hongsheng Chen, Ido Kaminer*, and Baile Zhang*

This PDF file includes:

-- Supplementary Methods

Section S1. Derivation of free-electron radiation from an interface

Section S2. Conventional formation time and formation zone

Section S3. Angular spectral energy density and energy spectral density of backward free-electron radiation

Section S4. More discussion on the time-domain study of the bulk-plasmon-mediated free-electron radiation from the vacuum-plasmonic medium interface

Section S5. History of Ferrell radiation

Section S6. Influence of the electron’s velocity on the long tail of bulk plasmons

Section S7. Influence of the nonlocal response of plasmonic media and the longitudinal waves on the bulk-plasmon-mediated free-electron radiation

-- Supplementary References

-- Caption for Supplementary Video

Supplementary Methods

Section S1. Derivation of free-electron radiation from an interface

By following Ginzburg and Frank's theory of transition radiation,[7-9,12] below we analytically derive the radiation process when a swift electron perpendicularly crosses an interface. The basic setup is the same as that shown in Figure 1 in the main text.

The current density for a swift particle with a charge q and a velocity $\bar{v} = \hat{z}v$ is given as

$$\bar{J}^q(\bar{r},t) = \hat{z}qv\delta(x)\delta(y)\delta(z-vt). \#(1)$$

We define the coordinate perpendicular to the boundary as $\bar{r}_\perp = \hat{x}x + \hat{y}y$. The current density is decomposed into a series of the function of frequencies and wave vectors via Fourier transform,

$$\bar{J}^q(\bar{r},t) = \hat{z}\int j^q_{\bar{\kappa}_{\perp,\omega}}(z)e^{i(\bar{\kappa}_\perp\cdot\bar{r}_\perp-\omega t)}d\bar{\kappa}_\perp d\omega, \#(2)$$

where $\bar{\kappa}_\perp = \hat{x}\kappa_x + \hat{y}\kappa_y$. From Equation (1-2), one gets $j^q_{\bar{\kappa}_{\perp,\omega}}(z) = \frac{q}{(2\pi)^3}\exp\left(i\frac{\omega}{v}z\right)$. Moreover, the electromagnetic fields can also be decomposed by Fourier transform,

$$\bar{E}(\bar{r},t) = \int \bar{E}_{\bar{\kappa}_{\perp,\omega}}(z)e^{i(\bar{\kappa}_\perp\cdot\bar{r}_\perp-\omega t)}d\bar{\kappa}_\perp d\omega,$$

$$\bar{H}(\bar{r},t) = \int \bar{H}_{\bar{\kappa}_{\perp,\omega}}(z)e^{i(\bar{\kappa}_\perp\cdot\bar{r}_\perp-\omega t)}d\bar{\kappa}_\perp d\omega. \#(3)$$

Since all the quantities are decomposed, the indices $\bar{\kappa}_\perp$ and ω are omitted for the sake of simplicity. By solving Maxwell equation, one can obtain the expression for the component of the electric field parallel to the particle's trajectory,

$$\frac{\partial^2}{\partial z^2}(\varepsilon_\mathrm{r}E_z) + \varepsilon_\mathrm{r}\left(\frac{\omega^2}{c^2}\varepsilon_\mathrm{r} - \kappa_\perp^2\right)E_z = -\frac{iq\omega\mu_0}{(2\pi)^3}\left(\varepsilon_\mathrm{r} - \frac{c^2}{v^2}\right)\exp\left(i\frac{\omega}{v}z\right), \#(4)$$

where ε_r is the relative permittivity and c is the speed of light in the free space. Only the TM (p-polarized) wave is excited in the studied structures in this work. The solution for Equation (4) is a linear sum of the field induced by the moving charge (E_z^q) and the freely radiated field (E_z^R). One may obtain the solution as

$$E_z^q = -\frac{iq}{\omega\varepsilon_0(2\pi)^3}\frac{1-\frac{c^2}{v^2\varepsilon_\mathrm{r}}}{\varepsilon_\mathrm{r} - \frac{c^2}{v^2} - \frac{\kappa_\perp^2c^2}{\omega^2}}e^{i\frac{\omega}{v}z}, \#(5)$$

$$E_z^R = \frac{iq}{\omega\varepsilon_0(2\pi)^3}\cdot a\cdot e^{\pm i\left(\frac{\omega}{c}\sqrt{\varepsilon_\mathrm{r}-\frac{\kappa_\perp^2c^2}{\omega^2}}\right)z}. \#(6)$$

where a is the amplitude of the radiation fields. As the radiation propagates away from the boundary, " $+$ " sign is used for region 2 ($z>0$) while "$-$" sign is used for region 1 ($z<0$). The value of a can be obtained by matching the boundary conditions below,

$$\hat{n}\times(\bar{H}_{1\perp}-\bar{H}_{2\perp})|_{z=0}=0,\qquad \hat{n}\times(\bar{E}_{1\perp}-\bar{E}_{2\perp})|_{z=0}=0,\#(7)$$

After some calculations, the amplitudes of the radiation fields in both regions are given as

$$a_{21}^{-}=\frac{\frac{v}{c}\frac{\kappa_\perp^2c^2}{\omega^2\varepsilon_{1\mathrm{r}}}(\varepsilon_{2\mathrm{r}}-\varepsilon_{1\mathrm{r}})\left[1-\frac{v^2}{c^2}\varepsilon_{1\mathrm{r}}+\frac{v}{c}\sqrt{\varepsilon_{2\mathrm{r}}-\frac{\kappa_\perp^2c^2}{\omega^2}}\right]}{\left(1-\frac{v^2}{c^2}\varepsilon_{1\mathrm{r}}+\frac{\kappa_\perp^2v^2}{\omega^2}\right)\left(1+\frac{v}{c}\sqrt{\varepsilon_{2\mathrm{r}}-\frac{\kappa_\perp^2c^2}{\omega^2}}\right)\left[\varepsilon_{1\mathrm{r}}\sqrt{\varepsilon_{2\mathrm{r}}-\frac{\kappa_\perp^2c^2}{\omega^2}}+\varepsilon_{2\mathrm{r}}\sqrt{\varepsilon_{1\mathrm{r}}-\frac{\kappa_\perp^2c^2}{\omega^2}}\right]},\#(8)$$

$$a_{12}^{+}=\frac{\frac{v}{c}\frac{\kappa_\perp^2c^2}{\omega^2\varepsilon_{2\mathrm{r}}}(\varepsilon_{2\mathrm{r}}-\varepsilon_{1\mathrm{r}})\left[1-\frac{v^2}{c^2}\varepsilon_{2\mathrm{r}}-\frac{v}{c}\sqrt{\varepsilon_{1\mathrm{r}}-\frac{\kappa_\perp^2c^2}{\omega^2}}\right]}{\left(1-\frac{v^2}{c^2}\varepsilon_{2\mathrm{r}}+\frac{\kappa_\perp^2v^2}{\omega^2}\right)\left(1-\frac{v}{c}\sqrt{\varepsilon_{1\mathrm{r}}-\frac{\kappa_\perp^2c^2}{\omega^2}}\right)\left[\varepsilon_{1\mathrm{r}}\sqrt{\varepsilon_{2\mathrm{r}}-\frac{\kappa_\perp^2c^2}{\omega^2}}+\varepsilon_{2\mathrm{r}}\sqrt{\varepsilon_{1\mathrm{r}}-\frac{\kappa_\perp^2c^2}{\omega^2}}\right]}.\#(9)$$

Note that a_{21}^{-} represents the backward radiation into region 1 while a_{12}^{+} represents the forward radiation into region 2. Other components of the electromagnetic fields can all be calculated from E_z. One can then get the expression for the electromagnetic field in the real space-time domain by integrating the solutions of Equation (5-6) concerning ω and $\kappa_\perp$. Since our model follows the cylindrical symmetry, the solution can be expressed in cylindrical coordinates (ρ,ϕ,z) for simplicity,

$$\bar{E}_1^q(\bar{r},t)=\hat{z}\int_{-\infty}^{+\infty}d\omega\frac{-q}{8\pi\omega\varepsilon_0\varepsilon_{1\mathrm{r}}}\left(\frac{\omega^2}{c^2}\varepsilon_{1\mathrm{r}}-\frac{\omega^2}{v^2}\right)H_0^{(1)}\left(\rho\sqrt{\frac{\omega^2}{c^2}\varepsilon_{1\mathrm{r}}-\frac{\omega^2}{v^2}}\right)e^{i\left(\frac{\omega}{v}z-\omega t\right)}$$
$$+\hat{\rho}\int_{-\infty}^{+\infty}d\omega\frac{-q}{8\pi\omega\varepsilon_0\varepsilon_{1\mathrm{r}}}\left(i\frac{\omega}{v}\right)\left(-\sqrt{\frac{\omega^2}{c^2}\varepsilon_{1\mathrm{r}}-\frac{\omega^2}{v^2}}\right)H_1^{(1)}\left(\rho\sqrt{\frac{\omega^2}{c^2}\varepsilon_{1\mathrm{r}}-\frac{\omega^2}{v^2}}\right)e^{i\left(\frac{\omega}{v}z-\omega t\right)},\#(10)$$

$$\bar{H}_1^q(\bar{r},t)=\hat{\phi}\int_{-\infty}^{+\infty}d\omega\frac{iq}{8\pi}\sqrt{\frac{\omega^2}{c^2}\varepsilon_{1\mathrm{r}}-\frac{\omega^2}{v^2}}H_1^{(1)}\left(\rho\sqrt{\frac{\omega^2}{c^2}\varepsilon_{1\mathrm{r}}-\frac{\omega^2}{v^2}}\right)e^{i\left(\frac{\omega}{v}z-\omega t\right)},\#(11)$$

$$\bar{E}_1^R(\bar{r},t)=\hat{z}\int_{-\infty}^{+\infty}d\omega\int_0^{+\infty}d\kappa_\perp\frac{iq}{(2\pi)^3\omega\varepsilon_0}a_{21}^{-}\kappa_\perp\big(2\pi J_0(\kappa_\perp\rho)\big)e^{i\left[-\left(\frac{\omega}{c}\sqrt{\varepsilon_{1\mathrm{r}}-\frac{\kappa_\perp^2c^2}{\omega^2}}\right)z-\omega t\right]}$$
$$+\hat{\rho}\int_{-\infty}^{+\infty}d\omega\int_0^{+\infty}d\kappa_\perp\frac{iq}{(2\pi)^3\omega\varepsilon_0}a_{21}^{-}\left(\frac{\omega}{c}\sqrt{\varepsilon_{1\mathrm{r}}-\frac{\kappa_\perp^2c^2}{\omega^2}}\right)\big(i2\pi J_1(\kappa_\perp\rho)\big)\,e^{i\left[-\left(\frac{\omega}{c}\sqrt{\varepsilon_{1\mathrm{r}}-\frac{\kappa_\perp^2c^2}{\omega^2}}\right)z-\omega t\right]},\#(12)$$

$$\bar{H}_1^R(\bar{r},t)=\hat{\phi}\int_{-\infty}^{+\infty}d\omega\int_0^{+\infty}d\kappa_\perp\frac{iq}{(2\pi)^3\omega\varepsilon_0}a_{21}^{-}(-\omega\varepsilon_{1\mathrm{r}}\varepsilon_0)\big(i2\pi J_1(\kappa_\perp\rho)\big)e^{i\left[-\left(\frac{\omega}{c}\sqrt{\varepsilon_{1\mathrm{r}}-\frac{\kappa_\perp^2c^2}{\omega^2}}\right)z-\omega t\right]},\#(13)$$

$$\bar{E}_2^q(\bar{r},t) = \hat{z}\int_{-\infty}^{+\infty} d\omega \frac{-q}{8\pi\omega\varepsilon_0\varepsilon_{2r}}\left(\frac{\omega^2}{c^2}\varepsilon_{2r} - \frac{\omega^2}{v^2}\right) H_0^{(1)}\left(\rho\sqrt{\frac{\omega^2}{c^2}\varepsilon_{2r} - \frac{\omega^2}{v^2}}\right) e^{i\left(\frac{\omega}{v}z-\omega t\right)}$$

$$+\hat{\rho}\int_{-\infty}^{+\infty} d\omega \frac{-q}{8\pi\omega\varepsilon_0\varepsilon_{2r}}\left(i\frac{\omega}{v}\right)\left(-\sqrt{\frac{\omega^2}{c^2}\varepsilon_{2r} - \frac{\omega^2}{v^2}}\right) H_1^{(1)}\left(\rho\sqrt{\frac{\omega^2}{c^2}\varepsilon_{2r} - \frac{\omega^2}{v^2}}\right) e^{i\left(\frac{\omega}{v}z-\omega t\right)}, \#(14)$$

$$\bar{H}_2^q(\bar{r},t) = \hat{\phi}\int_{-\infty}^{+\infty} d\omega \frac{iq}{8\pi}\sqrt{\frac{\omega^2}{c^2}\varepsilon_{2r} - \frac{\omega^2}{v^2}}\, H_1^{(1)}\left(\rho\sqrt{\frac{\omega^2}{c^2}\varepsilon_{2r} - \frac{\omega^2}{v^2}}\right) e^{i\left(\frac{\omega}{v}z-\omega t\right)}, \#(15)$$

$$\bar{E}_2^R(\bar{r},t) = \hat{z}\int_{-\infty}^{+\infty} d\omega \int_0^{+\infty} d\kappa_\perp \frac{iq}{(2\pi)^3\omega\varepsilon_0} a_{12}^{+}\kappa_\perp\big(2\pi J_0(\kappa_\perp\rho)\big) e^{i\left[+\left(\frac{\omega}{c}\sqrt{\varepsilon_{2r}-\frac{\kappa_\perp^2 c^2}{\omega^2}}\right)z-\omega t\right]}$$

$$+\hat{\rho}\int_{-\infty}^{+\infty} d\omega \int_0^{+\infty} d\kappa_\perp \frac{iq}{(2\pi)^3\omega\varepsilon_0} a_{12}^{+}\left(-\frac{\omega}{c}\sqrt{\varepsilon_{2r} - \frac{\kappa_\perp^2 c^2}{\omega^2}}\right)\big(i2\pi J_1(\kappa_\perp\rho)\big)\, e^{i\left[+\left(\frac{\omega}{c}\sqrt{\varepsilon_{2r}-\frac{\kappa_\perp^2 c^2}{\omega^2}}\right)z-\omega t\right]}, \#(16)$$

$$\bar{H}_2^R(\bar{r},t) = \hat{\phi}\int_{-\infty}^{+\infty} d\omega \int_0^{+\infty} d\kappa_\perp \frac{iq}{(2\pi)^3\omega\varepsilon_0} a_{12}^{+}(-\omega\varepsilon_{2r}\varepsilon_0)\big(i2\pi J_1(\kappa_\perp\rho)\big) e^{i\left[+\left(\frac{\omega}{c}\sqrt{\varepsilon_{2r}-\frac{\kappa_\perp^2 c^2}{\omega^2}}\right)z-\omega t\right]}. \#(17)$$

<u>Section S2. Conventional formation time and formation zone</u>

For transition radiation, according to Ginzburg's work,[8,9] the length of the formation zone is defined as the distance that the charge field and the radiation field separate from each other. In other words, the contribution of the interference term $E^q \cdot E^R$ to the total field energy (proportional to $|E^q + E^R|^2$) must be very small outside the formation zone. According to Equation (5-6), the charge field and radiation field for the backward radiation will have a minor interference if[8,9]

$$\left[\frac{\omega}{v} + \frac{\omega}{c}\sqrt{\varepsilon_{1r} - \frac{\kappa_\perp^2 c^2}{\omega^2}}\right] \cdot z \gg 2\pi. \#(18)$$

Namely, when the interference term oscillates rapidly, its integration over the real space is small. Therefore, the formation length L_{f1} in region 1 and L_{f2} in region 2 are defined as[8,9]

$$L_{f1,2} = \frac{2\pi}{\left|\frac{\omega}{v} \pm \frac{\omega}{c}\sqrt{\varepsilon_{1r,2r} - \frac{\kappa_\perp^2 c^2}{\omega^2}}\right|}, \#(19)$$

where the " $+$ " sign is used for region 1 while the "$-$ " sign is used for region 2. Accordingly, the formation length t_{f1} in region 1 and t_{f2} in region 2 is defined as $t_{f1,2} = L_{f1,2}/v$, or

$$t_{f1,2} = \frac{2\pi/v}{\left|\frac{\omega}{v} \pm \frac{\omega}{c}\sqrt{\varepsilon_{1r,2r} - \frac{\kappa_\perp^2 c^2}{\omega^2}}\right|}. \#(20)$$

In this work, we are interested in the backward-radiation fields emitted at small angles (namely $\kappa_\perp \approx 0$) at the frequency very close to the plasma frequency. This way, we denote in the main text $t_\mathrm{f}(\omega) = \frac{2\pi}{\omega\left|1+\frac{v}{c}\sqrt{\varepsilon_{1\mathrm{r}}}\right|} + \frac{2\pi}{\omega\left|1-\frac{v}{c}\sqrt{\varepsilon_{2\mathrm{r}}}\right|}$ as the conventional formation time of transition radiation (whose related fields propagate parallel to the electron's trajectory). To facilitate related discussions, we adopt the conventional formation time $t_{\mathrm{f}0} = t_\mathrm{f}(\omega_\mathrm{p}) = \frac{2\pi}{\omega_\mathrm{p}\left|1+\frac{v}{c}\right|} + \frac{2\pi}{\omega_\mathrm{p}\left|1-\frac{v}{c}\sqrt{\varepsilon_{2\mathrm{r}}}\right|}$ (or the formation length $L_{\mathrm{f}0} = t_{\mathrm{f}0}v$) as a reference to normalize related parameter in all related figure plots.

Section S3. Angular spectral energy density and energy spectral density of backward free-electron radiation

Analytical derivation of angular spectral energy density and energy spectral density

One can calculate the total energy of emitted photons in the backward free-electron radiation with a simple approach by considering the radiation fields at $t \to \infty$.[12,29,45] The backward radiation energy in region 1 (or free space) is expressed as

$$W_1 = \int dr_\perp \int_0^{+\infty} \varepsilon_1 \, |\bar{E}_1^R(\bar{r},t)|^2 \, dz, \#(21)$$

where $|\bar{E}_1^R(\bar{r},t)|^2 = \int\int\int\int \bar{E}^R_{1|\bar{\kappa}_\perp,\omega}(z) \cdot \bar{E}^{R^*}_{1|\bar{\kappa}'_\perp,\omega'}(z) \exp(i[(\bar{\kappa}_\perp - \bar{\kappa}'_\perp)\cdot \bar{r}_\perp - (\omega-\omega')t])\, d\bar{\kappa}_\perp d\bar{\kappa}'_\perp d\omega d\omega'$, it is the modulus square of the radiation field. Two expressions are used for the integration over the space,

$$\int dr_\perp e^{i(\bar{\kappa}_\perp - \bar{\kappa}'_\perp)\bar{r}_\perp} = (2\pi)^2 \delta(\kappa_\perp - \kappa'_\perp), \#(22)$$

$$\int \exp\left(i\frac{\omega}{c}\sqrt{\varepsilon_{1\mathrm{r}} - \frac{\kappa_\perp^2 c^2}{\omega^2}} - i\frac{\omega'}{c}\sqrt{\varepsilon_{1\mathrm{r}} - \frac{\kappa_\perp^2 c^2}{\omega'^2}} \right) z\, dz = \frac{2\pi c}{\varepsilon_{1\mathrm{r}}}\sqrt{\varepsilon_{1\mathrm{r}} - \frac{\kappa_\perp^2 c^2}{\omega^2}}\,\delta(\omega-\omega'). \#(23)$$

Furthermore, note that $|E^R_{1\perp}|^2 = \frac{\omega^2}{\kappa_\perp^2 c^2}\left(\varepsilon_{1\mathrm{r}} - \frac{\kappa_\perp^2 c^2}{\omega^2}\right)|E^R_{1z}|^2$, the expression for radiation energy is obtained as

$$W_1 = 2\int_0^{+\infty}\int \frac{q^2 \varepsilon_{1\mathrm{r}}}{(2\pi)^3 \varepsilon_0 c \kappa_\perp^2}\sqrt{\varepsilon_{1\mathrm{r}} - \frac{\kappa_\perp^2 c^2}{\omega^2}}\,|a_1|^2 d\bar{\kappa}_\perp d\omega\,. \#(24)$$

We recall that $\kappa_\perp = k_1 \sin\theta = \frac{\omega}{c}\sqrt{\varepsilon_{1\mathrm{r}}}\sin\theta$, then the integration of $d\bar{\kappa}_\perp$ is expanded as

$$d\bar{\kappa}_\perp = d\kappa_x d\kappa_y = 2\pi\kappa_\perp d\kappa_\perp = 2\pi\frac{\omega^2}{c^2}\varepsilon_{1\mathrm{r}}\sin\theta\cos\theta\, d\theta. \#(25)$$

The final expression for the total backward radiation energy is given as

$$W_1 = \int_0^{+\infty} W_1(\omega)\, d\omega. \qquad (26\text{-}1)$$

The energy spectral density $W_1(\omega)$ of backward radiation is denoted as

$$W_1(\omega) = \int_0^{\pi/2} (2\pi \sin\theta) U_1(\omega,\theta)\, d\theta \tag{26-2}$$

The angular spectral energy density $U_1(\omega,\theta)$ of backward radiation is written as

$$\begin{aligned} U_1(\omega,\theta) &= \frac{\varepsilon_{1r}^{3/2} q^2 \cos^2\theta}{4\pi^3 \varepsilon_0 c \sin^2\theta} |a_1|^2 \\ &= \frac{\varepsilon_{1r}^{3/2} q^2 \beta^2 \cos^2\theta \sin^2\theta}{4\pi^3 \varepsilon_0 c |1-\varepsilon_{1r}\beta^2\cos^2\theta|^2} \left| \frac{(\varepsilon_{2r}-\varepsilon_{1r})\left[1-\beta^2\varepsilon_{1r}+\beta\sqrt{\varepsilon_{2r}-\varepsilon_{1r}\sin^2\theta}\right]}{\left(1+\beta\sqrt{\varepsilon_{2r}-\varepsilon_{1r}\sin^2\theta}\right)\left[\varepsilon_{1r}\sqrt{\varepsilon_{2r}-\varepsilon_{1r}\sin^2\theta}+\varepsilon_{2r}\sqrt{\varepsilon_{1r}} cos\theta\right]} \right|^2 \end{aligned} \tag{26-3}$$

where $\beta = \frac{v}{c}$ and c is the speed of light in free space.

The angular distribution for the backward free-electron radiation, such as that in Figure 4, can be plotted by using Equation (26). As another example, Figure S1 shows the angular spectral energy density of backward free-electron radiation (at a frequency close to the plasma frequency) for the time-domain study in Figure 2a-b.

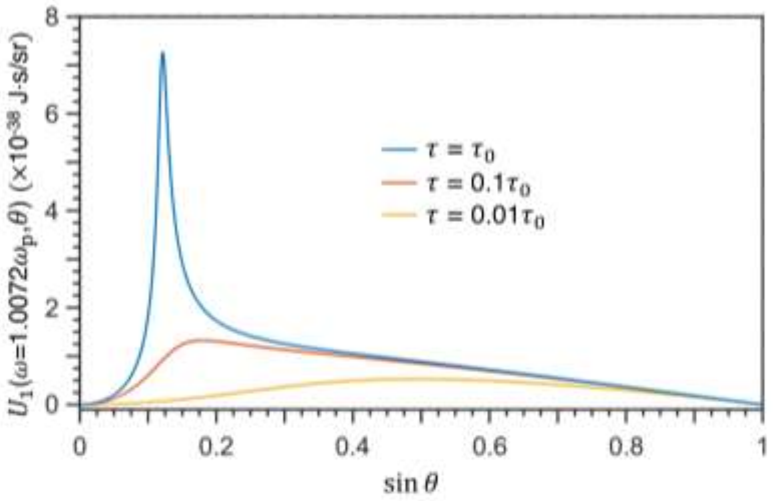

Figure S1. Angular spectral energy density for the time-domain study of backward free-electron radiation in Figure 2a-b and Figure S4 below. Here the working frequency is set to be $\omega = 1.0072\omega_p$. Region 2 is the plasmonic medium with the relaxation time τ being τ_0 (the value used in Figure 2a-c), $0.1\tau_0$ and $0.01\tau_0$ (these two values are adopted for comparison; see also Figure S4), respectively. When $\tau = \tau_0$, there is a peak at $\theta \approx 7°$. This peak is related to the bulk-plasmon-mediated free-electron radiation beyond the conventional formation time in Figure 2a-b. To capture the features of bulk-plasmon-mediated free-electron radiation, we study the dynamical evolution of the backward radiation field at a point $\bar{r}_{far}$ far away from the interface but close to the electron's trajectory in Figure 2a-b, where the angle between $\bar{r}_{far}$ and $-\bar{z}$ is $\theta_{far} \approx 7°$.

As complementary information to Figure 4a and to see the influence of material loss, we plot in Figure S2 the angular spectral energy density of backward free-electron radiation from the interface of the vacuum-plasmonic medium with different values of relaxation time τ for the plasmonic medium.

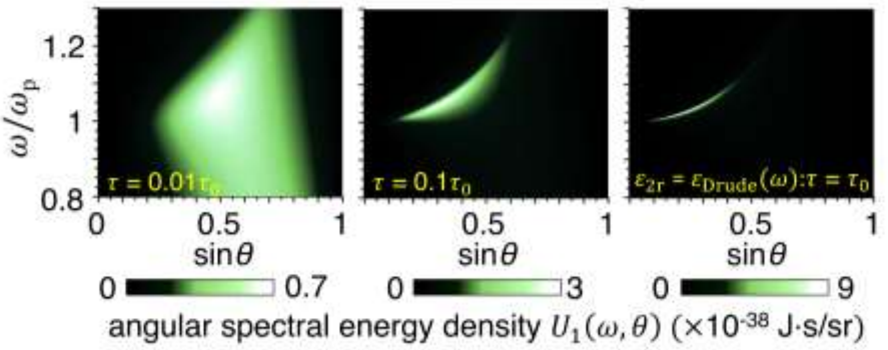

Figure S2. More discussion on the angular spectral energy density $U_1(\omega, \theta)$ with $\varepsilon_{2r} = \varepsilon_{Drude}(\omega)$ in Figure 4a by using various values of τ. There is a radiation peak at each frequency (near the plasma frequency). The radiation peak has a relatively broad angular distribution if τ is small (e.g., θ ranges from 15° to 60° if $\tau = 0.01\tau_0$). If τ increases, the radiation peak becomes to have an enhanced magnitude and narrower angular distribution; see also Figure 4a.

More discussion of Figure 4a

This sub-section serves as the complementary information for Figure 4a. The bulk plasmons have a frequency around ω_p, while the surface plasmons supported by the metal interface only exist below the frequency of $\omega_p/\sqrt{2}$. This way, the bulk plasmons and surface plasmons are independent, and they will not interact with each other. Actually, the surface plasmons are mainly formed within the conventional formation time of transition radiation. On the other hand, due to the momentum mismatch, the excited surface plasmons cannot couple into the vacuum. As a result, there is no radiation peak at the frequency (e.g. around $\omega_p/\sqrt{2}$) where surface plasmons exist, as can be seen from the backward angular spectral energy density in Figure S3.

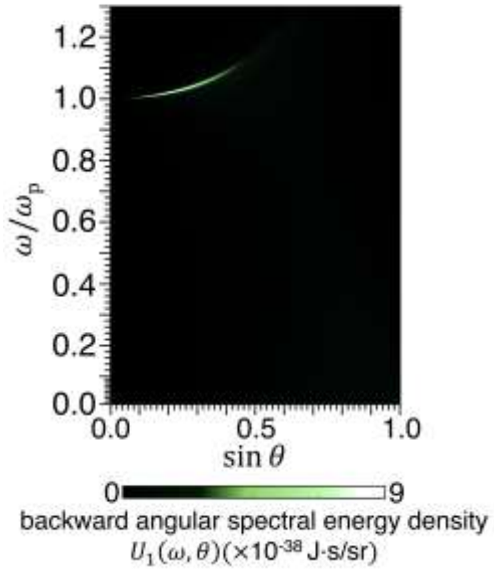

Figure S3. Angular spectral energy density of backward radiation. All setups are the same as Figure 4a. This figure serves as the complementary information for Figure 4a. While Figure 4a is plotted from $0.8\omega_\mathrm{p}$ to $1.3\omega_\mathrm{p}$, this figure is plotted in a wider frequency range, namely from 0 to $1.3\omega_\mathrm{p}$. From this figure, there is no radiation peak below the frequency of $\omega_\mathrm{p}/\sqrt{2}$, below which the surface plasmons are supported by the metal interface.

Section S4. More discussion on the time-domain study of the bulk-plasmon-mediated free-electron radiation from the vacuum-plasmonic medium interface

More frequency-domain analysis of the bulk-plasmon-mediated free-electron radiation in Figure 2a-c & S1

As complementary information for Figure 2a-c and Figure S1 and to see the loss influence on the free-electron radiation, here we show more analysis of the free-electron radiation by setting $\tau = 0.1\tau_0$ & $0.01\tau_0$ in Figure S4. From the time-domain and frequency-domain analyses in Figure 2a-c, Figure S1 and Figure S4, after the electron crosses the interface, the radiation beyond the conventional formation time is highly dependent on the value of τ (or the material loss), especially for the frequency component near the plasma frequency. In contrast, the radiation within the conventional formation time (which can be roughly estimated as the difference between the two lines in the frequency domain in Figure 2c and Figure S4c & f) is slightly affected by τ. This way, it is reasonable to argue that the free-electron radiation from the interface of plasmonic media within and beyond the conventional formation time is governed by different physical mechanisms. Namely, the free-electron radiation within the conventional formation time is the

same as the transition radiation from regular dielectrics, and in contrast, the free-electron radiation beyond the conventional formation time is mainly due to the interaction between the long tail of bulk plasmons inside the plasmonic medium with the interface as shown in Figure 3. We thus denote the radiation within and beyond the conventional formation time as the conventional free-electron radiation (or transition radiation) and the bulk-plasmon-mediated free-electron radiation in this work, respectively.

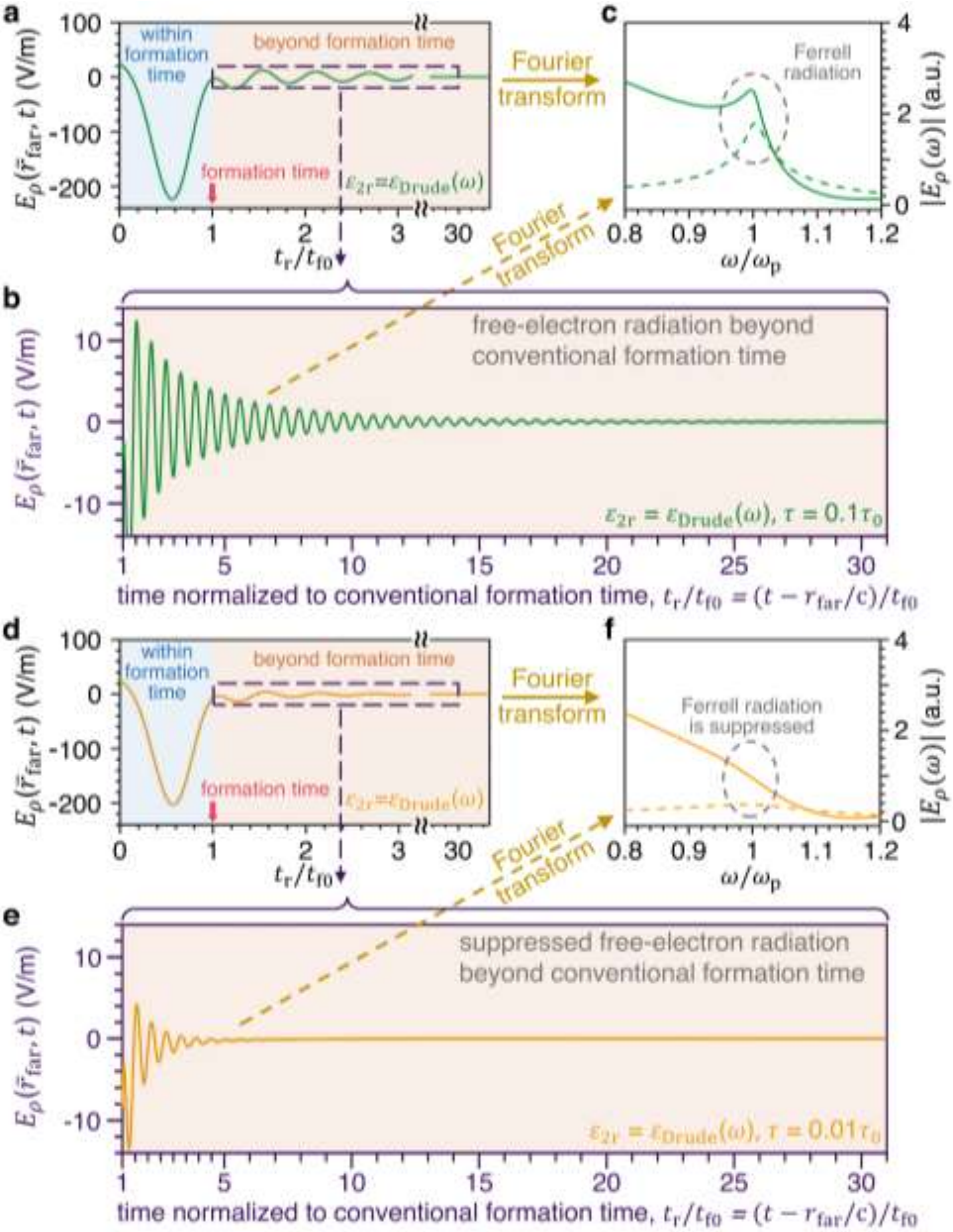

Figure S4. More analysis of the bulk-plasmon-mediated free-electron radiation in Figure 2a-c by setting $\boldsymbol{\tau = 0.1\tau_0}$ & $\boldsymbol{0.01\tau_0}$. All other setup is the same as Figure 2a-c. (**a-b**) Time-domain analysis if $\tau = 0.1\tau_0$ and (**d-e**) Time-domain analysis if $\tau = 0.01\tau_0$ The bulk-plasmon-mediated free-electron radiation beyond the conventional formation time will become more apparent if the material loss decreases or τ increases. Frequency domain analysis if (**c**) $\tau = 0.1\tau$ and (**f**) $\tau = 0.01\tau_0$. The peak of Ferrell radiation

near the plasma frequency in the frequency spectrum is mainly caused by the bulk-plasmon-mediated free-electron radiation beyond the conventional formation time. If τ decreases, the peak of Ferrell radiation becomes less obvious or even vanishes (compared with that in Figure 2c), due to the suppression of the bulk-plasmon-mediated free-electron radiation.

More analysis on the field distribution when the electron crosses an interface in Figure 3a-d

As complementary information for Figure 3a-d, the field distribution when a fast electron propagates in a homogeneous medium is shown in Figure S5; see the related discussion in the figure caption.

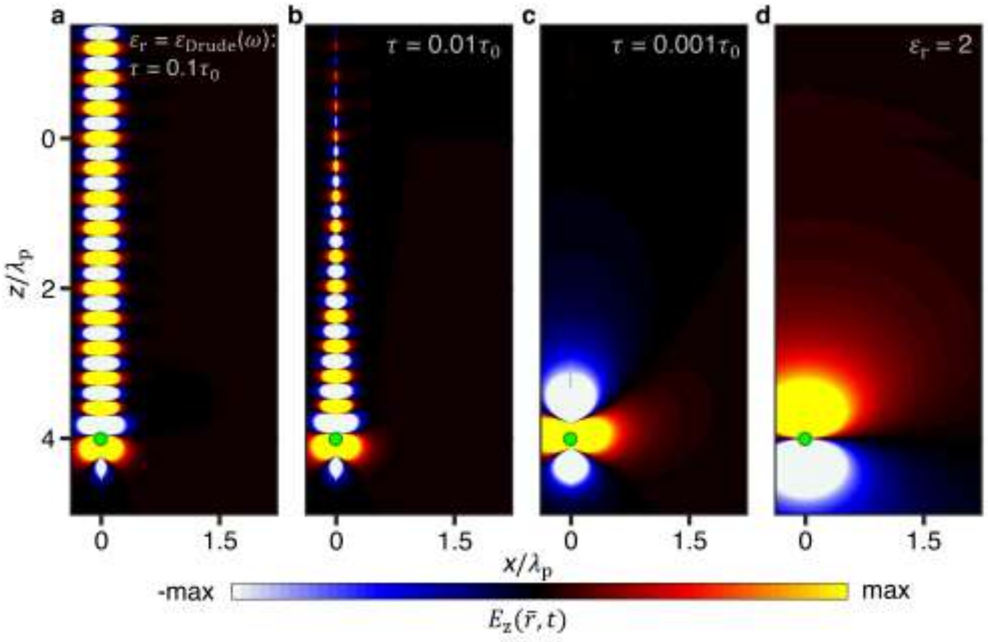

Figure S5. Field distribution when a fast electron moves in a homogeneous material. (**a-c**) The homogeneous material is a plasmonic medium with $\varepsilon_r = \varepsilon_{Drude}(\omega)$. (**d**) The homogeneous material is a dielectric with $\varepsilon_r = 2$. The long tail of bulk plasmons appears only when the electron moves inside a plasmonic medium with a relatively-large relaxation time τ, such as that in (a, b); the tail length increases with τ. The other structural setup is the same with Figure 3a-d.

Section S5. History of Ferrell radiation

We introduce in this section the interesting history of Ferrell radiation since it is fraught with controversies at the very beginning. In 1958, Ferrell firstly developed an approximate theory for the radiation of plasma oscillation by pointing out that “under suitable circumstances the plasma oscillations (which can be excited by swift electrons) will give off electromagnetic radiation”.[31] Due to the importance of Ferrell radiation in

the measurement of metal's plasma frequency,[31] the peak in the radiation spectrum for Ferrell radiation was soon confirmed in experiments in 1960.[33,34] However, Silin and Fetisov argued in 1961 that Ferrell radiation is just the transition radiation predicted by Ginzburg and Frank in 1945.[35] Moreover, they considered the nonlocal response of metal and pointed out that the bulk longitudinal plasma oscillation will not cause the radiation peak. Later in 1962, Stern explained that Ferrell's method and the theory of transition radiation "are two different ways to consider the same phenomenon".[38] Although "Ferrell's method only calculates the peak," it clearly "shows the physical mechanism causing the peak".[38] Stern further emphasized that Silin and Fetisov misinterpreted Ferrell's physical mechanism, which is not the bulk longitudinal plasma oscillation in their study but "a surface effect",[38] i.e., "the contribution of radiative surface plasma oscillation (SPO)".[39] (It shall be emphasized the electromagnetic fields related to the plasma oscillation mentioned in Ferrell's method[31] are transverse waves, instead of longitudinal waves.) However, Economou insisted in 1969[39] that "there are no radiative SPO in the present geometry (i.e., a thin metal slab)" and preferred the explanation related to the transition radiation; to be specific, his explanation mainly relies on the mathematical analysis of "the denominator in the expression of transition radiation near the peak" of Ferrell radiation. [39] With the rapid development of plasmonics, it is now argued that the radiative SPO is essentially a leaky or radiative mode in the studied system and is termed as the Ferrell mode.[32,38,40,44]

As emphasized in the main text, Ferrell's approximate theory and the theory of transition radiation indeed provide two seemingly distinct underlying mechanisms for Ferrell radiation, namely regarding whether it is a surface or bulk effect, from transition radiation or plasmonic oscillation, and so far there is no decisive conclusion. With our revealed mechanism of bulk-plasmon-mediated free-electron radiation beyond the conventional formation time, it becomes feasible to settle this historical debate here; see the discussion in the main text.

Section S6. Influence of the electron's velocity on the long tail of bulk plasmons

When a swift electron moves inside a plasmonic medium with a minor material loss, the long tail of bulk plasmons will appear, as shown in Figure 3. The bulk-plasmon tail revealed here (namely its related electromagnetic field) is transverse in nature and different from the longitudinal bulk plasmons.[35,38] To

address the issue related to the longitudinal bulk plasmons, one needs to consider the nonlocal response of the plasmonic medium, which will be discussed in the next section.

Due to its transverse nature, the bulk-plasmon tail follows the dispersion relation of transverse electromagnetic waves in the plasmonic medium, i.e., $\kappa_\perp^2 + \frac{\omega^2}{v^2} = \frac{\omega^2}{c^2}\varepsilon_{\mathrm{Drude}}$. Since $v < \mathrm{c}/\sqrt{\varepsilon_{\mathrm{Drude}}}$ at the frequency close to the plasma frequency, $\kappa_\perp$ will be an imaginary number (if the loss is neglected), and the bulk-plasmon tail will decay in the $\bar{r}_\perp$-direction (or in the direction perpendicular to the electron's trajectory). In other words, the bulk-plasmon tail is always well confined in space and follows the electron's trajectory inside the plasmonic medium.

Figure S6 shows that the appearance of the bulk-plasmon tail is irrelevant to the particle velocity, but its shape will change with the particle velocity. Due to its almost periodical oscillating property in the direction of particle's motion, the tail has a wavelength in the $\bar{z}$-direction, which is around $\lambda_{\mathrm{z,tail}} = 2\pi v/\omega_{\mathrm{p}}$. Importantly, $\lambda_{\mathrm{z,tail}}$ increases with v, as shown in Figure S6.

Moreover, the bulk-plasmon-mediated free-electron radiation from the plasmonic medium is also studied for different electron's velocities in Figure S7. Figure S7a shows that the angular spectral energy density of backward radiation increases with the electron's velocity, but the angular position of its peak is insensitive to the electron's velocity. Figure S6b&c illustrates the time evolution of the radiated field at a point $\bar{r}_{\mathrm{far}}$ far away from the boundary at $\theta = 7^\circ$. We note that the appearance of the bulk-plasmon-mediated free-electron radiation beyond the conventional formation time has a minor dependence on the electron's velocity.

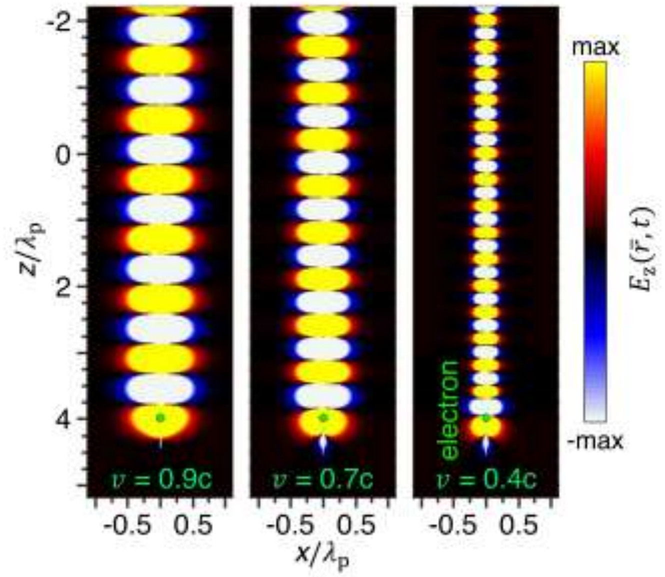

Figure S6. Long tail of bulk plasmons when a swift electron moves inside a homogeneous plasmonic medium with different velocities. The relaxation time of the plasmonic medium is $\tau = 0.1\tau_0$. The bulk-plasmon tail is well confined near to the electron's trajectory, follows the electron's motion, and has a wavelength $\lambda_{\mathrm{z,tail}}$ in the $\bar{z}$-direction, where $\lambda_{\mathrm{z,tail}} \to 2\pi v/\omega_{\mathrm{p}}$ and $\lambda_{\mathrm{z,tail}}$ increases with v.

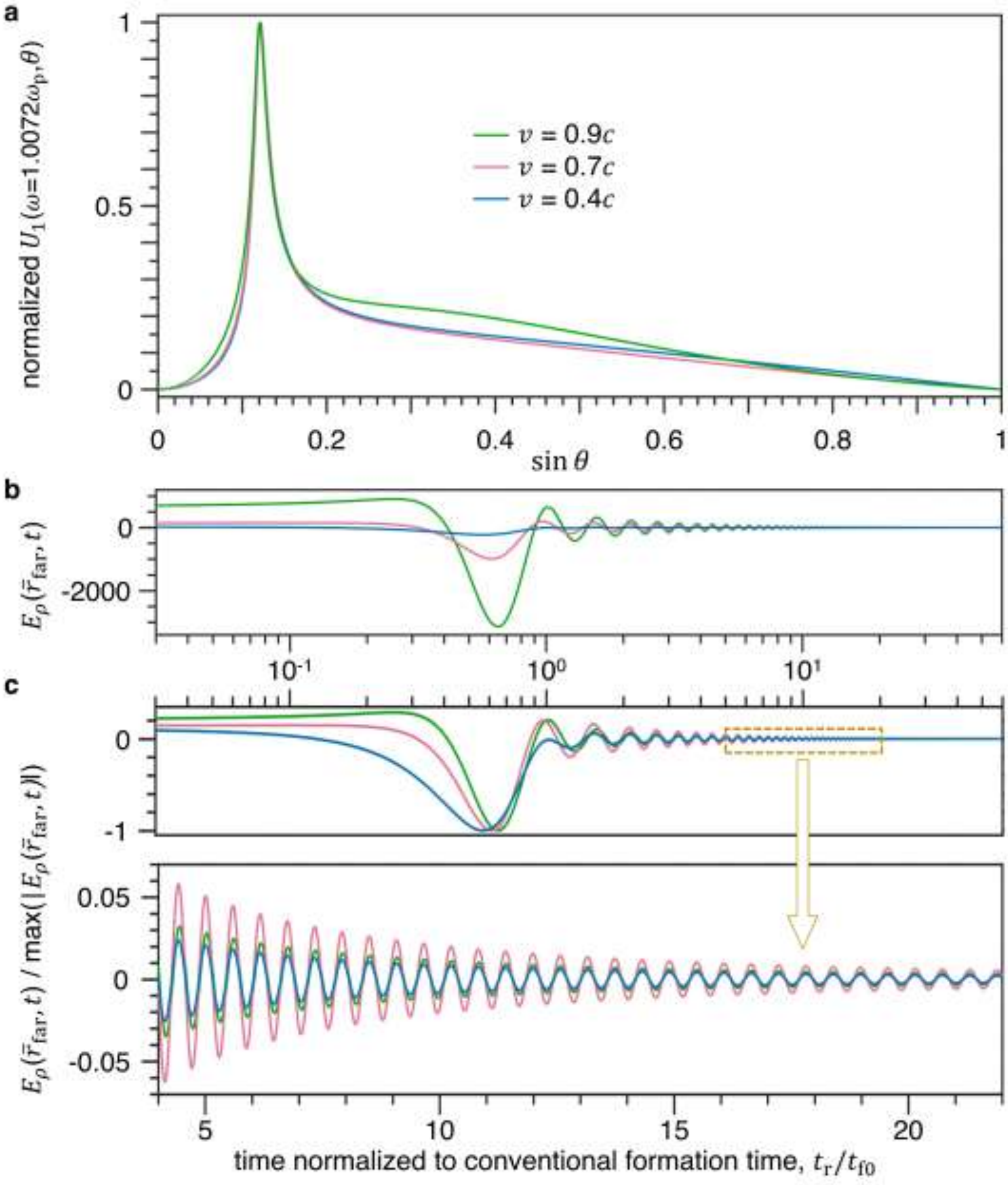

Figure S7. Bulk-plasmon-mediated free-electron radiation under different electron's velocities. A swift electron, moving with different velocities, v, across an interface between vacuum and the plasmonic medium. The basic setup is the same as Figure 2a-b. (**a**) Angular spectral energy density of backward radiation at a frequency close to the plasma frequency. (**b**) Dynamical evolution of radiated field at a far point $\bar{r}_{\mathrm{far}}$ in vacuum, where the angle between $\bar{r}_{\mathrm{far}}$ and $-\bar{z}$ is $\theta = 7°$. (**c**) Time evolution of the normalized

radiation field from (b). The appearance of bulk-plasmon-mediated free-electron radiation has a minor dependence on the electron's velocity. For the plasmonic medium, we set $\tau = \tau_0$. For the sake of clarity, the horizontal axis of all plots here are normalized by the same t_{f0}, namely the conventional formation time calculated by setting $v = 0.4c$.

Section S7. Influence of the nonlocal response of plasmonic media and the longitudinal waves on the bulk-plasmon-mediated free-electron radiation

Derivation of free-electron radiation with the consideration of nonlocal response and longitudinal waves

When a plasmonic medium (e.g., noble metals) has its relative permittivity close to zero, the longitudinal wave might appear. As a general discussion, the longitudinal wave can be considered by including the nonlocal response or spatial dispersion of plasmonic media in our model. Below we address the influence of the nonlocal response of plasmonic media and the longitudinal waves on our revealed phenomena, by studying the angular spectral energy density of backward radiation in the frequency domain and the dynamical evolution of the radiation field in the time domain.

To consider the nonlocal response of the plasmonic medium, we adopt the hydrodynamic model.[46-51] In the hydrodynamic model, the permittivity of the medium exhibits different values for the transverse and longitudinal waves. Accordingly, the Maxwell equation is modified into

$$\left(k^2 - \frac{\omega^2}{c^2}\varepsilon^T\right)\bar{E}^T - \frac{\omega^2}{c^2}\varepsilon^L\bar{E}^L = -i\omega\mu_0(\bar{J}^T + \bar{J}^L), \#(27)$$

where T and L in the superscripts denote the transverse and longitudinal components. It is worthy to note that $k^2 = \kappa_\perp^2 + k_z^2$, $\bar{k} \times \left(\bar{k} \times \bar{E}^T\right) = k^2 E^T$ for transverse waves and $\bar{k} \times \bar{E}^L = 0$ for longitudinal waves. By following a similar procedure in section S1 to solve the differential equation, the expressions for the charge field and the radiation field, for both transverse and longitudinal waves, are obtained as

$$E_z^{q(L)} = -\frac{iq\exp\left(i\frac{\omega}{v}z\right)}{\omega\varepsilon_0(2\pi)^3\left(\kappa_\perp^2 + \frac{\omega^2}{v^2}\right)}\frac{\left(\frac{\omega^2}{v^2}\right)}{\varepsilon^L}, \#(28)$$

$$E_z^{q(T)} = \frac{iq\exp\left(i\frac{\omega}{v}z\right)}{\omega\varepsilon_0(2\pi)^3\left(\kappa_\perp^2 + \frac{\omega^2}{v^2}\right)}\left[\frac{\kappa_\perp^2\frac{\omega^2}{c^2}}{\left(\kappa_\perp^2 + \frac{\omega^2}{v^2} - \frac{\omega^2}{c^2}\varepsilon^T\right)}\right], \#(29)$$

$$E_z^{R(L)} = \frac{iq}{\omega\varepsilon_0(2\pi)^3}a^L\exp(ik_z^L z), \#(30)$$

$$E_z^{R(T)} = \frac{iq}{\omega\varepsilon_0(2\pi)^3} a^T \exp(ik_z^T z) . \#(31)$$

The transverse wave satisfies the dispersion relation of $k^2 = \frac{\omega^2}{c^2}\varepsilon^T$, and thus $k_z^T = \sqrt{\frac{\omega^2}{c^2}\varepsilon^T - \kappa_\perp^2}$. On the other hand, the longitudinal wave satisfies the dispersion relation that $\varepsilon^L = 0$,[50] where

$$\varepsilon^L(\omega, k) = 1 - \frac{\omega_\mathrm{p}^2}{\omega^2 + i\omega/\tau - \beta_L^2 k^2}, \#(32)$$

In Equation (32), $\beta_L = \sqrt{3/5}\, v_F$ is the hydrodynamic wave vector, and k is the wave vector of the waves. This way, $k_z^L = \sqrt{k_{L0}^2 - \kappa_\perp^2}$ is related to the longitudinal permittivity, where k_{L0} is the solution of $\varepsilon^L = 0$. Next, the radiation fields in different regions, both for the transverse and longitudinal waves, can be solved by enforcing the boundary conditions. An additional boundary condition (ABC) is required to solve all unknowns. In short, the boundary conditions can be expressed as follows,

$$\left(E_{1z}^q + E_{1z}^R\right)|_{z=0} = \left(E_{2z}^{q(L)} + E_{2z}^{R(L)} + E_{2z}^{q(T)} + E_{2z}^{R(T)}\right)|_{z=0}, \#(33)$$

$$\left(H_{1\phi}^q + H_{1\phi}^R\right)|_{z=0} = \left(H_{2\phi}^{q(T)} + H_{2\phi}^{R(T)}\right)|_{z=0}, \#(34)$$

$$\left(\kappa_\perp E_{1\perp}^q + \kappa_\perp E_{1\perp}^R\right)|_{z=0} = \left(\kappa_\perp E_{2\perp}^{q(L)} + \kappa_\perp E_{2\perp}^{R(L)} + \kappa_\perp E_{2\perp}^{q(T)} + \kappa_\perp E_{2\perp}^{R(T)}\right)|_{z=0}. \#(35)$$

Since region 1 is composed of a regular dielectric, all fields in region 1 are transverse. After cumbersome calculations, the amplitude for the backward radiation in region 1 is obtained as

$$a_{21}^- = \frac{1}{\left[\frac{\omega}{c}\left(\varepsilon_{1\mathrm{r}}\sqrt{\varepsilon_{2\mathrm{r}}^T - \frac{\kappa_\perp^2 c^2}{\omega^2}} + \varepsilon_{2\mathrm{r}}^T\sqrt{\varepsilon_{1\mathrm{r}} - \frac{\kappa_\perp^2 c^2}{\omega^2}}\right) + \frac{\kappa_\perp^2}{k_{z2}^L}\left(\varepsilon_{1\mathrm{r}} - \varepsilon_{2\mathrm{r}}^T\right)\right]} \{ \frac{\kappa_\perp^2 \varepsilon_{2\mathrm{r}}^T \frac{\omega}{v}\left(\varepsilon_{2\mathrm{r}}^{L(q)} - \varepsilon_{2\mathrm{r}}^T\right)}{\varepsilon_{2\mathrm{r}}^{L(q)}\left(\kappa_\perp^2 + \frac{\omega^2}{v^2}\right)\left(\varepsilon_{2\mathrm{r}}^T - \frac{c^2}{v^2} - \frac{\kappa_\perp^2 c^2}{\omega^2}\right)}$$
$$+ \frac{\frac{\kappa_\perp^2 c^2}{\omega^2}\left(\varepsilon_{2\mathrm{r}}^T \frac{\omega}{v} - \varepsilon_{2\mathrm{r}}^{L(q)} \frac{\omega}{c}\sqrt{\varepsilon_{2\mathrm{r}}^T - \frac{\kappa_\perp^2 c^2}{\omega^2}}\right)}{\varepsilon_{2\mathrm{r}}^{L(q)}\left(\varepsilon_{2\mathrm{r}}^T - \frac{c^2}{v^2} - \frac{\kappa_\perp^2 c^2}{\omega^2}\right)} - \frac{\frac{\kappa_\perp^2 c^2}{\omega^2}\left(\varepsilon_{2\mathrm{r}}^T \frac{\omega}{v} - \varepsilon_{1\mathrm{r}} \frac{\omega}{c}\sqrt{\varepsilon_{2\mathrm{r}}^T - \frac{\kappa_\perp^2 c^2}{\omega^2}}\right)}{\varepsilon_{1\mathrm{r}}\left(\varepsilon_{1\mathrm{r}} - \frac{c^2}{v^2} - \frac{\kappa_\perp^2 c^2}{\omega^2}\right)}$$
$$+ \frac{\kappa_\perp^2}{k_{z2}^L}\left[\frac{\varepsilon_{2\mathrm{r}}^T\left(\varepsilon_{2\mathrm{r}}^{L(q)}\kappa_\perp^2 + \varepsilon_{2\mathrm{r}}^T \frac{\omega^2}{v^2}\right)}{\varepsilon_{2\mathrm{r}}^{L(q)}\left(\kappa_\perp^2 + \frac{\omega^2}{v^2}\right)\left(\varepsilon_{2\mathrm{r}}^T - \frac{c^2}{v^2} - \frac{\kappa_\perp^2 c^2}{\omega^2}\right)} - \frac{\varepsilon_{2\mathrm{r}}^{L(q)}\frac{\kappa_\perp^2 c^2}{\omega^2} + \varepsilon_{2\mathrm{r}}^T \frac{c^2}{v^2}}{\varepsilon_{2\mathrm{r}}^{L(q)}\left(\varepsilon_{2\mathrm{r}}^T - \frac{c^2}{v^2} - \frac{\kappa_\perp^2 c^2}{\omega^2}\right)} + \frac{\varepsilon_{1\mathrm{r}}\frac{\kappa_\perp^2 c^2}{\omega^2} - \varepsilon_{2\mathrm{r}}^T\left(\varepsilon_{1\mathrm{r}} - \frac{c^2}{v^2}\right)}{\varepsilon_{1\mathrm{r}}\left(\varepsilon_{1\mathrm{r}} - \frac{c^2}{v^2} - \frac{\kappa_\perp^2 c^2}{\omega^2}\right)}\right]\}. \#(36)$$

One should bear in mind that $\varepsilon_{2\mathrm{r}}^{L(q)}(\omega, k) = 1 - \frac{\omega_\mathrm{p}^2}{\omega^2 + i\omega/\tau - \beta_L^2(\omega^2/v^2 + \kappa_\perp^2)}$ in Equation (36), where $k^2 = \kappa_\perp^2 + \omega^2/v^2$ and ω/v is the $\bar{z}$-component of wavevector for the charge field.

Influence of the nonlocal response and longitudinal waves on the field distribution of free-electron radiation

Figure S7 studies the influence of the nonlocal response of the plasmonic medium and the excitation of longitudinal waves on the field distribution of free-electron radiation. We find the nonlocal response have a minor influence on the long tail of bulk plasmons inside the plasmonic medium and the radiation fields inside the region of free space; see Figure S8a-c. Therefore, it is reasonable to argue that the nonlocal response have a negligible influence on the bulk-plasmon-mediated free-electron radiation revealed in this work; see more analysis in Figure S9. Moreover, in addition to the long tail of transverse bulk plasmons, if we consider the nonlocal response, the Cherenkov radiation of longitudinal waves will also be excited when the electron moves inside the plasmonic medium; see Figure S8d-e. Since the excited longitudinal waves inside the plasmonic medium propagate forward (i.e., away from the interface) and cannot propagate far, their appearance also has a minor influence on the bulk-plasmon-mediated free-electron radiation revealed in this work.

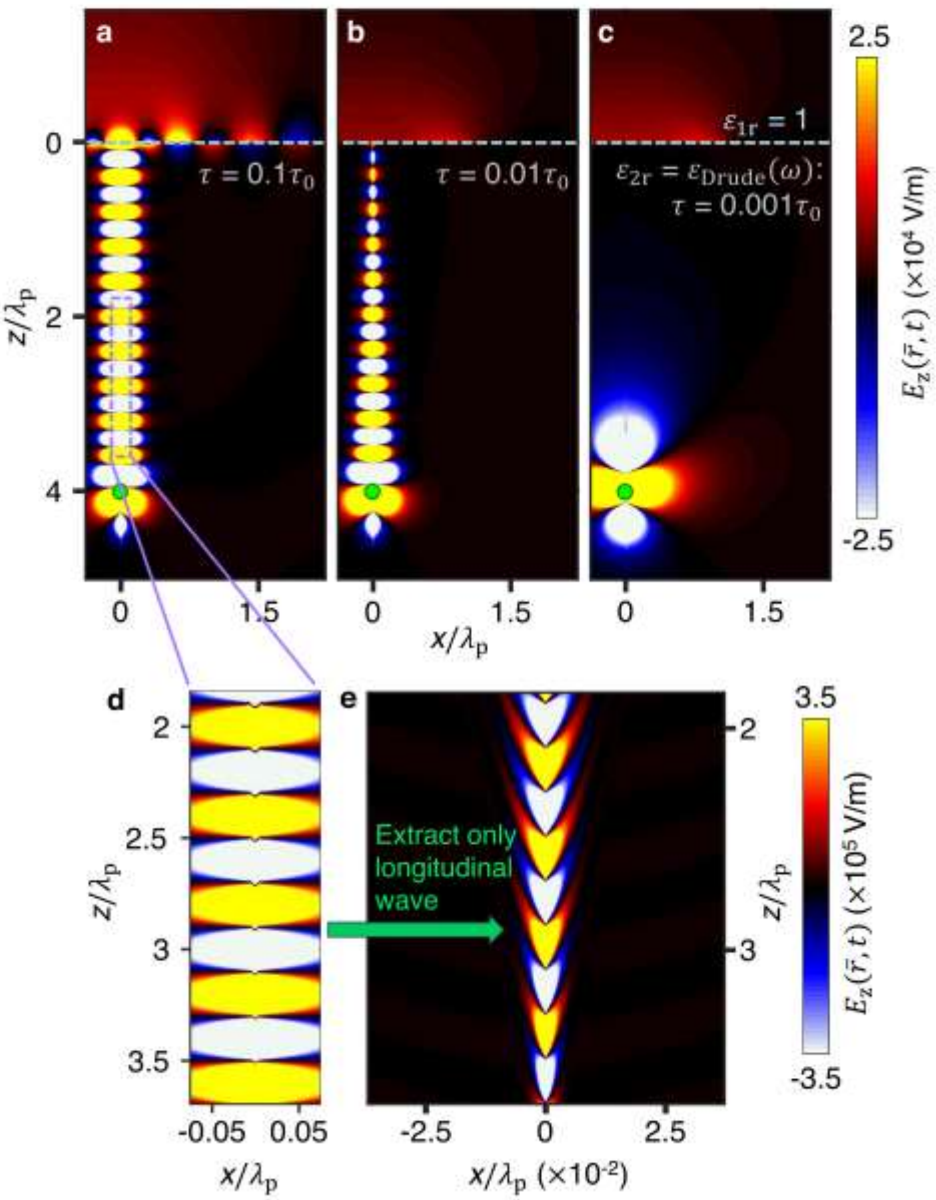

Figure S8. Influence of the nonlocal response and longitudinal waves on the distribution of total field when the electron perpendicularly crosses an interface of the vacuum-plasmonic medium. (a-c) Distribution of the total field with the same structural setup as Figure 3a-c and S4. **(d)** The magnified view of a small region is highlighted by the dashed square in (a). The plots in (a-d) contain both longitudinal and transverse waves. **(e)** Extraction of the longitudinal wave from (d); in other words, the plot in (e) only contains the longitudinal wave. While Figure 3a-c and S4 have neglected the nonlocal response of plasmonic media, here we study its influence. The nonlocal response of β_L is assumed to be $c/300$ (the value generally obtained from silver). Compared with Figure 3a-c, Figure S8a-c shows that the nonlocal response of plasmonic media has a minor influence on the radiation fields in the region of free space and the long tail of bulk plasmons inside the plasmonic medium. These figures indicate that the nonlocal

response has little influence on the radiation spectrum of backward radiation and the revealed bulk-plasmon-mediated free-electron radiation; see more in Figure S9. Besides, if we consider the nonlocal response, there will be some eigenmodes of longitudinal waves inside the plasmonic medium. This way, in addition to the long tail of transverse bulk plasmons mentioned in Figure 3a and S4a, there will also be the emergence of Cherenkov radiation of plane-like longitudinal waves inside the plasmonic medium. The magnitude of longitudinal waves is relatively weak and they (see the little spikes in the long tail of bulk plasmons in Figure S8d) are flooded by the long tail of transverse bulk plasmons; as a result, the longitudinal waves cannot be easily seen in the total field plots in Figure S8a-c. To visualize the excited longitudinal waves clearly, we plot them solely in Figure S8e. The excited longitudinal waves propagate away from the interface. Due to the material loss, they cannot propagate over a long distance or far away from the electron trajectory.

Influence of the nonlocal response and longitudinal waves on the angular spectral energy density and the bulk-plasmon-mediated free-electron radiation

Figure S9a demonstrates the influence of the nonlocal response of plasmonic media and the excitation of longitudinal waves on the angular spectral energy density of backward radiation from the interface of the vacuum-plasmonic medium. Although the nonlocal response slightly affects the magnitude of the peak, the peak is still pronounced in Figure S9a. Furthermore, the time-domain study of bulk-plasmon-mediated free-electrons radiation in Figure S9b confirms the minor influence of the nonlocal response on the bulk-plasmon-mediated free-electron radiation.

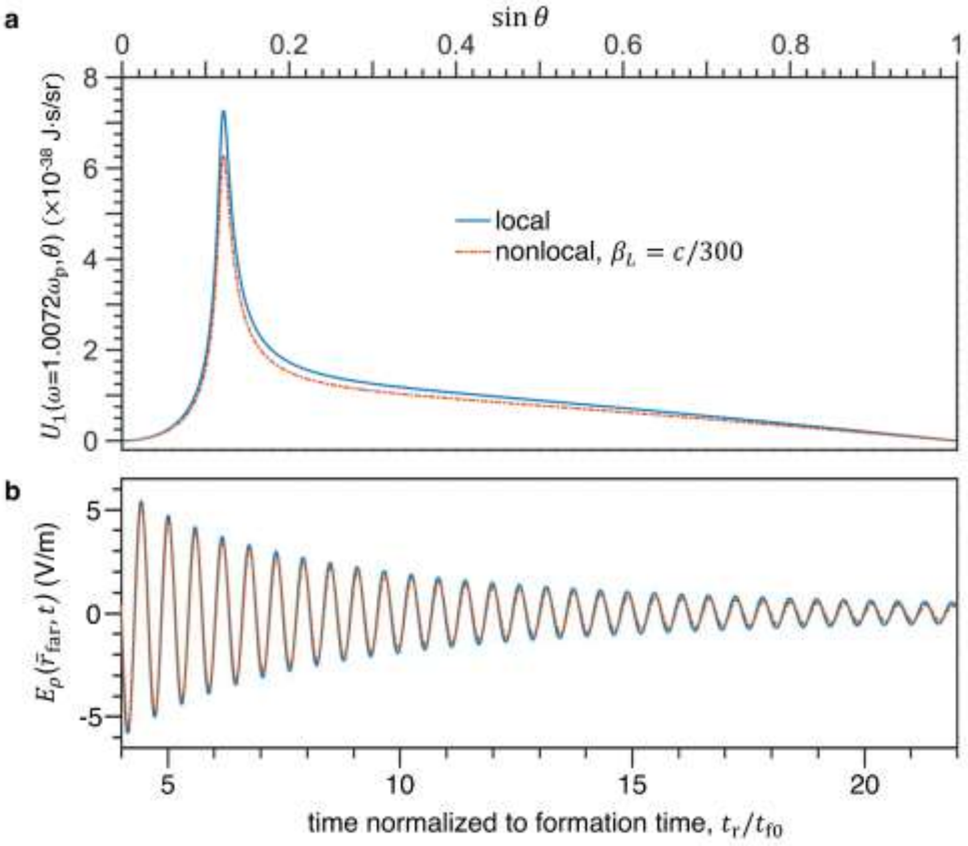

Figure S9. Influence of nonlocal response on the bulk-plasmon-mediated free-electron radiation. A swift electron passes an interface between free space and a plasmonic medium. The basic setup is the same as Figure 2 and the plasmonic medium has $\tau = \tau_0$. (**a**) Angular spectral energy density of backward radiation at a frequency close to the plasma frequency. (**b**) Time evolution of the radiated field at a point far from the interface , where the angle between $\bar{r}_{\text{far}}$ and $-\bar{z}$ is $\theta = 7°$. The hydrodynamic wave vector β_L characterizes the nonlocal response, where the nonlocal response increases with β_L. As a typical example, $\beta_L = c/300$ (which is the one used for the study of the nonlocal response of silver) is adopted. The nonlocal response has a minor influence on the bulk-plasmon-mediated free-electron radiation.

Supplementary References

[45] L. D. Landau, E. M. Lifshitz, L. P. Pitaevskii, *Electrodynamics of Continuous Media*, Pergamon, **1984**.

[46] S. Raza, G. Toscano, A.-P. Jauho, M. Wubs, N. A. Mortensen, *Phys. Rev. B* **2011**, *84*, 121412.

[47] A. A. Orlov, P. M. Voroshilov, P. A. Belov, Y. S. Kivshar, *Phys. Rev. B* **2011**, *84*, 045424.

[48] W. Yan, M. Wubs, N. A. Mortensen, *Phys. Rev. B* **2012**, *86*, 205429.

[49] C. Ciracì, R. T. Hill, J. J. Mock, Y. Urzhumov, A. I. Fernández-Domínguez, S. A. Maier, J. B. Pendry, A. Chilkoti, and D. R. Smith, *Science* **2012**, *337*, 1072.

[50] Y. Luo, A. I. Fernandez-Dominguez, A. Wiener, S. A. Maier, and J. B. Pendry, *Phys. Rev. Lett.* **2013**, *111*, 093901.
[51] L. Ferrari, C. Wu, D. Lepage, X. Zhang, and Z. Liu, *Prog. Quantum Electron.* **2015**, *40*, 1.

Caption for Supplementary Video

Movie S1. Dynamical evolution of $E_z(\bar{r},t)$ field distribution in Figure 3a.

Movie S2. Dynamical evolution of $E_z(\bar{r},t)$ field distribution in Figure 3b.

Movie S3. Dynamical evolution of $E_z(\bar{r},t)$ field distribution in Figure 3c.

Movie S4. Dynamical evolution of $E_z(\bar{r},t)$ field distribution in Figure 3d.

Movie S5. Field distribution when a charge field moves inside a plasmonic medium with various values of relaxation time τ. The other structural setup is the same as Figure S5.
